# PLACD-7T Study: Atherosclerotic Carotid Plaque Components Correlated with Cerebral Damage at 7 Tesla Magnetic Resonance Imaging

**DOI:** 10.2174/157340311795677743

**Published:** 2011-02

**Authors:** A.G den Hartog, S.M Bovens, W Koning, J Hendrikse, G Pasterkamp, F.L Moll, G.J de Borst

**Affiliations:** 1 Departments of Vascular Surgery, Utrecht, the Netherlands, Interuniversity Cardiology Institute of the Netherlands (ICIN), Utrecht, The Netherlands; 2 Experimental Cardiology Utrecht, the Netherlands, Interuniversity Cardiology Institute of the Netherlands (ICIN), Utrecht, The Netherlands; 4 Radiology Utrecht, Utrecht, the Netherlands, Interuniversity Cardiology Institute of the Netherlands (ICIN), Utrecht, The Netherlands; 3 University Medical Center Utrecht, Utrecht, the Netherlands, Interuniversity Cardiology Institute of the Netherlands (ICIN), Utrecht, The Netherlands

**Keywords:** 7 Tesla MRI, carotid plaque, cerebral damage, histology.

## Abstract

**Introduction:**

In patients with carotid artery stenosis histological plaque composition is associated with plaque stability and with presenting symptomatology. Preferentially, plaque vulnerability should be taken into account in pre-operative work-up of patients with severe carotid artery stenosis. However, currently no appropriate and conclusive (non-) invasive technique to differentiate between the high and low risk carotid artery plaque *in vivo* is available. We propose that 7 Tesla human high resolution MRI scanning will visualize carotid plaque characteristics more precisely and will enable correlation of these specific components with cerebral damage.

**Study objective:**

The aim of the PlaCD-7T study is 1: to correlate 7T imaging with carotid plaque histology (gold standard); and 2: to correlate plaque characteristics with cerebral damage ((clinically silent) cerebral (micro) infarcts or bleeds) on 7 Tesla high resolution (HR) MRI.

**Design:**

We propose a single center prospective study for either symptomatic or asymptomatic patients with haemodynamic significant (70%) stenosis of at least one of the carotid arteries. The Athero-Express (AE) biobank histological analysis will be derived according to standard protocol. Patients included in the AE and our prospective study will undergo a pre-operative 7 Tesla HR-MRI scan of both the head and neck area.

**Discussion:**

We hypothesize that the 7 Tesla MRI scanner will allow early identification of high risk carotid plaques being associated with micro infarcted cerebral areas, and will thus be able to identify patients with a high risk of periprocedural stroke, by identification of surrogate measures of increased cardiovascular risk.

## INTRODUCTION

Ipsilateral carotid stenosis of 50% or more is found in approximately 10% of carotid territory ischemic strokes and approximately 15% of transient ischemic attacks (TIAs). Additionally, it is associated with a high risk of recurrent stroke [1, 2]. Current treatment of symptomatic patients with carotid stenosis is based on stenosis grade alone, where stenosis ≥70% indicates carotid endarterectomy (CEA). Plaque morphology is not yet included in this clinical decision, although it plays an important role as it is found to be directly correlated with the risk of embolism and progression to occlusion, thus resulting in cerebral ischaemia [3]. Thrombo-embolic events, caused by (a) (ruptured) atherosclerotic carotid plaque(s), can cause a TIA or ischemic stroke. Carotid plaque phenotype is a major independent determinant of the clinical outcome after CEA, while plaque composition is related to clinical presentation, gender, age and time interval between most ischemic symptoms and CEA [4-9].

Athero-Express [10] (AE; plaque biobank) studies have previously shown that certain compositions of the atherosclerotic plaque are associated with pre- and postoperative cerebral embolisation measured with Trans Cranial Doppler (TCD). Clinically, patients with a more inflamed plaque showed more embolisation [11], and the composition of the atherosclerotic plaque was associated with repeated cerebral infarcts after surgery [12]. In addition, patients with a fibrotic plaque had a lower risk for recurrent TIA or stroke than patients with a more lipid-rich plaque [3]. In the same study, during three years follow-up, it was found that a high rate of neovasculature with intra-plaque haemorrhage (IPH) was a strong predictor for cardiovascular events elsewhere in the vascular tree. Thus, the classically defined measures for plaque vulnerability (plaque bleeding, large lipid cores and local inflammation) were all related with adverse outcomes peri-operatively as well as during follow up.

Important intra-plaque markers for plaque stability are the relative amounts of smooth muscle cells (SMC) and collagen. Hellings *et al.* showed that women have more stable plaques compared to men, with specifically more stable plaques in asymptomatic women. These stable plaques were fibrotic and had a relatively higher SMC and collagen content [5].

Identification of high-risk patients, preferably with minimally invasive techniques such as MRI, could lead to different peri- and postoperative monitoring of CEA patients. MRI has the capability to visualize plaque characteristics in the stenotic carotid artery. Several preliminary studies, both *ex vivo* and *in vivo*, have been performed to assess the possibility of plaque imaging with MRI [13-20].

Ultimately we aim that high resolution (HR) 7 Tesla diagnostic accuracy will be valuable in clinical decision making by differentiating low risk and high risk patients and thereby determining which patients benefit most from revascularization. This prognostic study could ultimately lead to the addition of a 7 Tesla HR-MRI scan to differentiate low risk and high risk patients.

### *Ex Vivo* MRI Plaque Studies

In an *ex vivo* study using dissected human carotid arteries (autopsy material), plaque components were evaluated using different HR-MRI weightings at 9.4 Tesla (vertical Bruker system). The outcome of this study suggested that HR-MRI is able to visualize several plaque characteristics, which were previously related with increased embolisation and repeated stroke. Multi-weighted MRI sequences could identify calcified regions, lipid area, foam cell area, IPH, neovasculature and the fibrous cap (submitted data; te Boekhorst *et al.*). Although this study was performed on autopsy material (no flow effects of blood) and at a high magnetic field, it gives information about which components could be visualized with the 7 Tesla human MRI scanner, whereas this might be more challenging if not impossible in a lower field MRI scanner. Several other *ex vivo* studies on human MRI scanners (1.5 Tesla) have been performed on atherosclerotic plaques obtained from CEA [13, 14]. Both studies found sensitivity values ranging from 60-97% to 72-98% for fibrous tissue, necrosis, calcification and for loose connective tissue. Specificity varied from 75%-98% to 74%-99% for the same plaque characteristics.

### *In Vivo* MRI Plaque Studies

Several human *in vivo* studies investigated 1.5 and 3 Tesla MRI as a tool to accurately determine plaque components, sometimes with histology as a reference [15-20]. For example, one study revealed sensitivity and specificity of 84%, a negative predictive value of 70%, and a positive predictive value of 93% of direct thrombus imaging with MRI, with histology as the gold standard. This would suggest that MRI can accurately determine plaque components with an 1.5 Tesla MRI scanner [17]. However, these studies merit careful consideration. The resolution of the 1.5 Tesla scanner is very limited and although the 3 Tesla scanner already has a higher resolution, for example imaging of large IPH and thin/ruptured fibrous cap is possible [17], moderate IPH is difficult to detect. In addition, up to now it has not been possible to discriminate between IPH and intra-plaque lipid. The latter is of great interest since recent studies have shown that IPH and not intra-plaque lipid is related with adverse events during follow up [3].

In summary, we believe due to the limitations of the above reported study methods and lower resolution of the current MRI scanners, it is valuable to perform an *in vivo* study with the 7 Tesla scanner to optimize the relevance of plaque characterization. In the present article we describe the protocol of our study designed to determine the relation between carotid plaque characteristics and cerebral damage by 7 Tesla HR-MRI. To our knowledge this is the first study that investigates this relation with a 7 Tesla MRI scanner.

## STUDY OBJECTIVE

We plan to conduct a single center, prospective, observational study, using a 7 Tesla MRI scanner (Philips Healthcare, Cleveland, OH, USA). The primary objective of PlaCD-7T (atherosclerotic carotid PLAque components correlated with Cerebral Damage at 7 Tesla) is to compare 7T HR-imaging with carotid plaque histology (gold standard). Our secondary objective is to correlate plaque characteristics with cerebral damage ((clinically silent) cerebral (micro) infarcts or bleeds) on 7 Tesla HR-MRI.

Histology will be performed to compare the *ex* *vivo* specimen with the *in vivo* images. Only one pre-operative HR-MRI scan will be added to the standard work-up of CEA patients in our hospital.

### MRI 7.0 Tesla Scanner

**Fig. (2).**
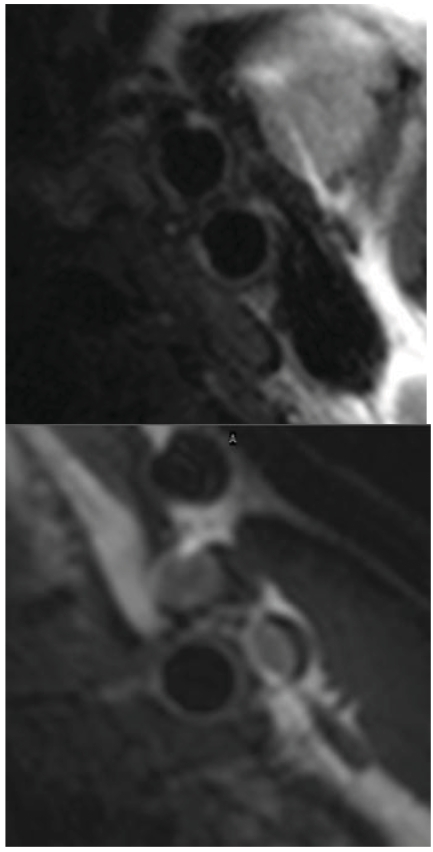
Imaging examples of the carotid artery on 7 Tesla MRI, acquired with a 4 channel tranceive coil (Machnet B.V., Eelde, The Netherlands).

7 Tesla MRI benefits from a high intrinsic Signal-to- Noise Ratio (SNR) compared to MRI with lower field strengths. The gain in SNR can be expressed in shorter scan times as well as higher resolution. Above that, different contrast mechanisms due to changed magnetic parameters in the tissues may lead to higher sensitivities for certain contrasts. For example, susceptibility differences are greater at 7 Tesla as well as the differences in chemical shift and relaxation times between different tissues. Also, higher resolution imaging suffers less from partial voluming effects, enabling the visualization of more detailed structures. More and more research is being done to explore this potential of 7 Tesla MRI [21, 22]. High field MR (plaque) imaging with 7 Tesla may allow plaque characterization and localization of cerebral damage with a higher resolution and more detail than imaging at lower field strengths. For instance, not only obvious cerebral ischemic lesions can be found, but also microbleeds, micro-infarcts and clinically silent ischemic lesions can be visualized, currently not possible with 1.5 Tesla or 3 Tesla MRI [22] (Fig. **2**).

The main reason for imaging patients scheduled for CEA at 7 Tesla is the high resolution and high contrast brain scan. Since we would like to correlate cerebral damage to specific plaque components it is of importance to have superior image quality of the brain. At the moment a few human studies at 7 Tesla have successfully been performed and published [23-26]. In addition, hypothetically, plaque imaging with 7 Tesla MRI could provide more detailed images of the plaque components. The 1.5 and 3 Tesla MRI may lack some discriminative power to assess plaque characteristics that correlate with risk for future manifestations of the disease. However, even if 7 Tesla MRI is not capable of discriminating more components in plaque compared to 3 Tesla MRI, the addition of the 7 Tesla brain imaging will provide new insights into the pathogenesis of cerebral damage as a result of plaque composition. Apart from these new insights, when this study shows that micro-infarctions, micro-embolisations or plaque characteristics can be visualized with these 7 Tesla MRI scans, then subsequent studies might be performed to establish the value of non-invasive MRI scanning in pre- operative work-up in patients with carotid artery stenosis.

## STUDY DESIGN

This is an observational study. First of all, patients with a stenosis ≥70% of the carotid artery will visit the vascular surgeon or neurologist in our hospital. Subsequently these patients are discussed in our multidisciplinary vascular meeting (vascular surgeons, neurologists, radiologists). When eligible for CEA, the patient will be included via the vascular surgeon in the AE study as well as the PlaCD-7T study. The majority of the patients will be symptomatic (TIA or stroke). However, a minority of asymptomatic patients with a haemodynamically significant stenosis >80% of the carotid artery and planned to be scheduled for a CEA will be eligible for inclusion in this study as well. Pre-operative (~1 day) the included patients are scheduled for a MRI scan of the brain and carotid artery in the 7 Tesla MRI scanner. Anatomical scans of the brain will be evaluated for the presence of cerebral damage. In the carotid artery, MRI images with several contrast weightings will be acquired for plaque visualization and characterization. A contrast agent (gadolinium (Gadovist®)) will be injected to assess neovascularisation, intraplaque haemorrhage and inflammation. This investigation will take approximately 75 minutes [27]. Images will be stored and during off line analyses the bifurcation of the carotid artery will be used as an anatomical landmark.

### Patient Population

All indications for surgery are reviewed by a multidisciplinary vascular team [10]. All patients are examined by a neurologist pre-operatively and postoperatively to document cerebrovascular symptom status and to record any new neurological deficits after CEA. Percentage stenosis of both carotid arteries is recorded with duplex ultrasound pre-operatively following internationally accepted guidelines [28]. A flowchart of the study protocol is shown in Fig. (**1**). Baseline characteristics of included patients will be collected by the AE investigators and include standard baseline characteristics together with known risk factors of cardiovascular disease. It is important to state that besides one MRI scan, patients will have the regular work-up, treatment, and follow-up according to the current guidelines for treatment of carotid artery disease.

### Athero-Express Biobank

AE is an ongoing longitudinal study that includes patients undergoing CEA. The study design has been reported earlier [10]. In our study gold standard for plaque characterization will be the histopathological analysis of the removed carotid artery plaque according to the standardized AE protocol. The segment with the largest plaque burden (the culprit lesion) is located and fixated in formalin for histological analysis. This analysis is routinely performed and intra- and inter-observer analyses are excellent and have been reported in international literature previously [27]. After surgery, the patients undergo normal clinical follow-up with duplex to assess procedural restenosis and to fill out questionnaires addressing the occurrence of relevant cardiovascular symptoms. All patients undergoing CEA in one participating center (University Medical Center Utrecht) will be asked to participate in this study. The medical ethics board of the University Medical Center Utrecht approved the study, and all participants of this study must provide written informed consent.

### Scan Protocol

**Fig. (3).**
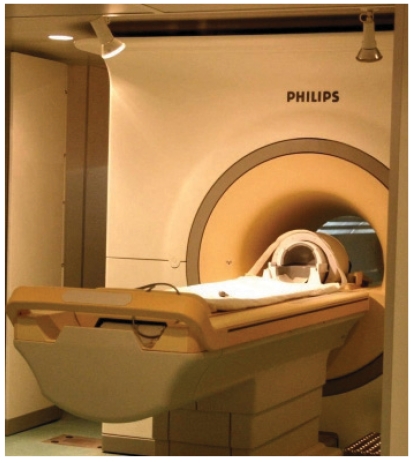
Procedural set-up in 7 Tesla scanner.

The total MRI examination including MR plaque imaging and anatomical MRI of the brain will take approximately 75 minutes (Fig. **3**). A dedicated RF coil is used for imaging of the carotid bifurcation. This coil allows for sub-millimeter resolution imaging of the carotid lumen, vessel wall and atherosclerotic plaque. First, the carotid bifurcation is identified by means of MR angiography without contrast enhancement. Subsequently, several transverse images are obtained from several millimeters caudal to several millimeters cranial of the carotid bifurcation, imaging the complete plaque. This area is imaged using specific sequences to obtain different contrast weightings for optimal discrimination of plaque components. Next, post-contrast images after intravenous injection of gadolinium will be obtained. Additionally, anatomical MR brain imaging of cerebral damage will be performed. For example, 3D FLAIR, T1W, SWI (susceptibility weighted imaging) and DWI (diffusion weighted imaging) images can be acquired using the head coil. Two MRI readers blinded to the histo-pathological results and clinical data will independently score the MRI plaque status.

### Carotid Endarterectomy

Pre-operatively, patients start with aspirin, except those patients already taking oral anticoagulants for other indications. For these patients dipyridamol 2dd200mg will be added. Patients with aspirin intolerance will receive plavix 75mg. Before exposure of the carotid artery, patients receive 5000 U of heparin intravenously. With the use of a standardized CEA technique, the plaque is carefully dissected and removed in toto, without procedure-related complications. Immediately after dissection, the plaque will be transferred to the laboratory.

### Histology

The atherosclerotic plaque will be decalcified in an EDTA solution, sliced at several loci, embedded in paraffin, and stained for presence of collagen (picrosirius red), macrophages (CD68), smooth muscle cells (-actin), lipid, thrombus and endothelium (CD34, microvessels or neovasculature). This analysis is routinely performed and intra- and inter-observer analyses are excellent and have been reported in international literature previously [27]. Histology is assessed by an experienced pathologist blinded to the MRI results.

### Sample Size

In a previous study we observed a >100% increase in peri-operative stroke when a lipid rich plaque was obtained compared to plaques that were fibrous [3]. A major endpoint was reached in 9% when a lipid rich lesion was evident but 2.5% when a fibrous lesion was present. We expect to see significantly more cerebral micro-infarcted areas (33% of patients). This data is based on the article of Verhoeven, *et al* [11]. In this study TCD was used to measure micro embolism. In 27% of the patients an event was registered before or during surgery. We expect the 7 Tesla system to be sensitive enough to find micro infarcts as a result of micro embolism. The power analysis is based on the calculation of the differences between two groups of a continuous variable. A 3-fold increase from 1 to 3 micro embolisms or 1 versus 3 micro infarcts with an SD of 2, an α of 0.05 and β of 0.80 we calculate that we will need 2 groups of 14 patients. Therefore we asked and received permission to include 30 patients. We do not expect a normal distribution.

### Data Analysis

MR images will be processed with dedicated software, with this software component size and intensity on images will be calculated. Histology will be semi-quantitatively scored as previously described [3, 10]. Cerebral damage ((clinically silent) cerebral (micro) infarcts or bleeds) will be depicted as a semi-quantitative measure (no, small, major defects). Micro-embolisation will be assessed as a continuous variable.

The different identified plaque components will be correlated with cerebral damage by calculating the regular parameters for diagnostic testing (positive predictive value, negative predictive value, sensitivity, specificity).

### Ethical Consideration

This study has been approved by the local Medical Ethical Committee of the University Medical Center of Utrecht.

## DISCUSSION

We present the protocol of a single center, observational study to determine the relation of plaque characteristics with cerebral damage ((clinically silent) cerebral (micro) infarcts or bleeds) with 7 Tesla HR-MRI. As gold standard histology of the plaques will be obtained according to the protocol of the AE biobank.

The interpretation of outcome in earlier studies imaging the carotid plaque and using histology as reference standard is complicated by the differences in histology methods, difference in the magnetic field strength of the MRI scanners and in scanning protocols (sequences).

In summary:

Characteristics *ex vivo* studies:

- No blood flow, thus no triggering is needed and no flow artifacts are seen. Additionally, artifacts caused by swallowing are also absent.
- The RF coil is placed very close to the plaque, creating higher signal intensities compared to *in vivo* measurements.

Limitations *ex vivo* studies:

Outcome can not always be extrapolated to the realistic *in vivo* measurements.

Limitations current *in vivo* studies:

- 1.5 Tesla and 3 Tesla scanners; lower resolution scanners
- not possible to discriminate between IPH and intraplaque lipid
- *in vivo*, the length of the neck and depth of the carotid artery are main determinants of image quality. Some sequences, which can give excellent contrast between components *ex vivo*, are not applicable *in vivo*, due to timing of the sequence or due to limited total scan time of the patient.
- different histology protocols

Advantages 7 Tesla MRI scanner:

- advanced brain imaging
- Higher resolution will reveal structures that are hidden by partial voluming and higher sensitivity for certain contrastmechanisms (e.g. susceptibility weighted imaging or Ultra short TE imaging (UTE)) will possibly allow detection of plaque components that are hard to detect with lower field strengths, like IPH).

Several studies have focused on the identification of atherosclerotic plaque components by means of MRI to identify patients at risk for strokes or TIAs [15-20]. Components such as large lipid pools, thin fibrous caps, and IPH are the main focus points due to the increased risk of plaque rupture. One of the components that is of importance and has been correlated with cardiovascular and cerebrovascular events is microvasculature in the plaque [3]. However, this component is very small (the same holds true for mild/moderate IPH) and needs an imaging modality with a high spatial resolution and anatomical detail such as the 7 Tesla scanner can provide. In addition, the 7 Tesla scanner is superior in the detection of micro-infacts and/or bleeds in the brain [26]. Combining this information with the HR-MRI of the atherosclerotic plaque, as described in this protocol, we believe this can give new insights and possibly lead to new standardized protocols for patient work-up before CEA.

In conclusion, we hypothesize that 7 Tesla HR-MRI will allow more detailed visualization of the brain, and is expected to determine the carotid plaque components more precisely. Therefore, in our opinion, this is the right modality to correlate brain imaging with carotid plaque characteristics. The AE biobank allows us to use histology as a validated reference according to standardized Athero-Express protocol.

The trial will start in March 2011 in the University Medical Center in Utrecht.

## TRIAL COLLABORATORS

Collaborators of this study (all located in the University Medical Center Utrecht, The Netherlands) are: (in alphabetical order):

G.J. de Borst, MD, PhD, vascular surgeon, S.M. Bovens, MSc, PhD student, A.G. den Hartog, MD, PhD student vascular surgery, J. Hendrikse, MD, PhD, radiologist, LJ Kappelle MD, PhD, Professor Neurology, D.J.W. Klomp, PhD, assistent professor Functional Medical Imaging, W. Koning, MSc, PhD student, P. Luijten, MD, PhD, Professor radiology, F.L. Moll, MD, PhD, Professor vascular surgery, G. Pasterkamp, MD, PhD, Professor experimental Cardiology, A. Vink, MD, PhD, pathologist, H.B. van der Worp, MD, PhD, neurologist J.J.M. Zwanenburg, PhD, post-doc.

## Figures and Tables

**Fig. (1).**
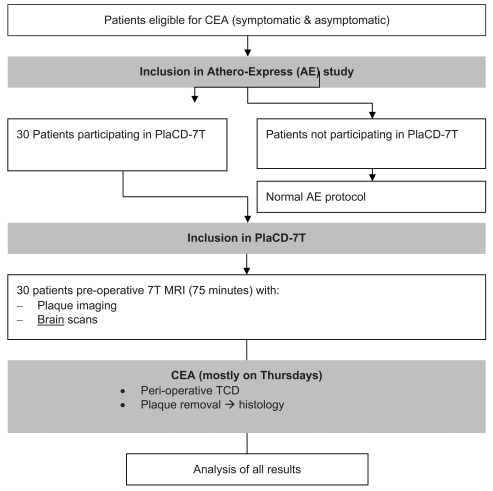
Simplified flowchart of study protocol.

## ABBREVIATIONS

CEA: = Carotid endarterectomy
HR: = High resolution
AE: = Athero-express
DWI: = Diffusion weighted imaging
IPH: = Intra-plaque haemorrhage
FLAIR: = Fluid attenuation inversion recovery
MRI: = Magnetic resonance imaging
RF: = Radio frequency
SMC: = Smooth muscle cells
SWI: = Susceptibility weighted imaging
T1W: = T1 weighted
TCD: = Trans Cranial Doppler
